# Effects of pulmonary acid aspiration on the lungs and extra-pulmonary organs: a randomized study in pigs

**DOI:** 10.1186/cc11214

**Published:** 2012-03-01

**Authors:** Jan Florian Heuer, Philip Sauter, Paolo Pelosi, Peter Herrmann, Wolfgang Brück, Christina Perske, Fritz Schöndube, Thomas A Crozier, Annalen Bleckmann, Tim Beißbarth, Michael Quintel

**Affiliations:** 1 Department of Anaesthesiology, Emergency and Intensive Care Medicine, University of Göttingen Medical Center, Robert-Koch-Straße 40, 37075 Göttingen, Germany; 2 Department of Surgical Sciences and Integrated Diagnostics, Univeristy of Genoa, L.go R. Benzi 10, 16132 Genoa, Italy; 3 Department of Neuropathology, University of Göttingen Medical Center, Robert-Koch-Straße 40, 37075 Göttingen, Germany; 4 Department of Pathology, University of Göttingen Medical Center, Robert-Koch-Straße 40, 37075 Göttingen, Germany; 5 Department of Thoracic- Heart- and Vascular Surgery, University of Göttingen Medical Center, Robert-Koch- Straße 40, 37075 Göttingen, Germany; 6 Department of Medical Statistics, University Medical Center Göttingen, Robert-Koch-Straße 40, 37075 Göttingen, Germany

## Abstract

**Introduction:**

There is mounting evidence that injury to one organ causes indirect damage to other organ systems with increased morbidity and mortality. The aim of this study was to determine the effects of acid aspiration pneumonitis (AAP) on extrapulmonary organs and to test the hypothesis that these could be due to circulatory depression or hypoxemia.

**Methods:**

Mechanically ventilated anesthetized pigs were randomized to receive intrabronchial instillation of hydrochloric acid (*n *= 7) or no treatment (*n *= 7). Hydrochloric acid (0.1 N, pH 1.1, 2.5 ml/kg BW) was instilled into the lungs during the inspiratory phase of ventilation. Hemodynamics, respiratory function and computer tomography (CT) scans of lung and brain were followed over a four-hour period. Tissue samples of lung, heart, liver, kidney and hippocampus were collected at the end of the experiment.

**Results:**

Acid instillation caused pulmonary edema, measured as increased extravascular lung water index (ELWI), impaired gas exchange and increased mean pulmonary artery pressure. Gas exchange tended to improve during the course of the study, despite increasing ELWI. In AAP animals compared to controls we found: a) cardiac leukocyte infiltration and necrosis in the conduction system and myocardium; b) lymphocyte infiltration in the liver, spreading from the periportal zone with prominent areas of necrosis; c) renal inflammation with lymphocyte infiltration, edema and necrosis in the proximal and distal tubules; and d) a tendency towards more severe hippocampal damage (*P *> 0.05).

**Conclusions:**

Acid aspiration pneumonitis induces extrapulmonary organ injury. Circulatory depression and hypoxemia are unlikely causative factors. ELWI is a sensitive bedside parameter of early lung damage.

## Introduction

Pulmonary aspiration of gastric contents is a recognized complication of anesthesia and emergency medicine that can cause acute lung failure and death [1-4]. Acid aspiration causes alveolar and interstitial inflammation with edema and leukocyte infiltration. Reduced alveolar gas content leads to increased venous admixture and pulmonary hypertension. The pathological consequences are not restricted to the lungs as the results of studies in small animals show. Acid aspiration can trigger a systemic inflammatory reaction with subsequent damage to other organs, such as heart, kidneys and brain [5-11]. This might explain, for example, why concurrent acute lung injury increases the mortality rate of acute renal failure by up to 80% [12]. The underlying mechanisms causing the extrapulmonary manifestations are not known. Among the proposed hypotheses are hypoxemia and/or hypoperfusion due to pulmonary dysfunction and circulatory depression [13] or damage mediated by a systemic inflammatory response induced by the pneumonitis [14]. Inapparent aspiration occurs under normal circumstances, and is found in 45% of healthy patients during sleep and in up to 70% of obtunded patients [15] with tachypnea and low-grade fever often being the only symptoms. This could have a significant adverse affect on morbidity and mortality in non-intubated intensive care patients.

The aim of this study was to investigate the early effects of acid-aspiration pneumonitis on function and morphology of the kidneys, heart, brain and liver in a large-animal model and to test whether hypoxemia or circulatory depression could be causative factors. The clinical relevance would be to further emphasize the importance of airway protection in reducing morbidity and mortality in intensive care patients.

## Materials and methods

### Experimental setting

The study had the approval of our institution's animal study review board (Bezirksregierung Braunschweig, Germany, Derzernat 604, Tierschutz). Care and handling of the animals were in accordance with the Helsinki and NIH guidelines.

Fourteen female domestic pigs (mean weight 58 kg; range 54 to 62 kg) were premedicated with 40 mg i.m. azaperonium. An ear vein was cannulated and Ringer acetate was infused at an average rate of 3 to 4 ml kg^-1 ^h^-1^. Anesthesia was induced with 3 to 5 mg·kg^-1 ^i.v. thiopental and 4 mg·kg^-1 ^i.v. ketamine and maintained with ketamine (10 mg kg^-1 ^h^-1^) and midazolam (1 mg kg^-1 ^h^-1^). A cuffed endotracheal tube was inserted and the lungs were ventilated (Servo 300, Siemens, Erlangen, Germany) in a volume-controlled, lung protective mode (positive end expiratory pressure (PEEP) 5 cm H_2_O; inspiratory:expiratory ratio I:E = 1:2; FiO_2 _= 1.0; respiratory rate 15 to 20 minutes^-1^; tidal volume V_T _= 8 ml kg^-1^). Respiratory rate was adjusted to maintain PaCO_2 _below 60 mmHg. End-tidal CO_2 _was monitored with a capnograph (Datex Capnomac Ultima, Helsinki, Finland).

Peripheral oxygen saturation, ECG and non-invasive blood pressure were monitored. continuously.

A Licox^® ^transcranial bolt was inserted through a burr hole in the right frontal region. A Licox^® ^microcatheter for intracranial pressure (ICP) (Integra Neuroscience, Integra GmbH, Ratingen, Germany) was inserted through the bolt into the white matter. The tip of this microprobe was placed approximately 25 mm below the dura.

A thermistor-tipped fiberoptic catheter (Pulsiocath, 4F FT PV 2024; Pulsion Medical System, Munich, Germany) was placed in a femoral artery. A pulmonary catheter (Volef, Pulsion Medical System, Munich, Germany) was inserted through a 5F sheath introduced in the right internal jugular vein and the position of the catheter tip confirmed by the pressure tracing. The catheters were connected to pressure transducers and to an integrated bedside monitor (PiCCO, Volef; Pulsion Medical Systems). After instrumentation, the animals were placed in the prone position for the rest of the study and baseline values were recorded.

#### Experimental protocol

The animals were randomly allotted to one of two groups: 1) acid aspiration pneumonitis (AAP) and 2) Control (Control).

##### Acid aspiration pneumonitis (AAP)

AAP was induced by intrabronchial installation of hydrochloric acid (HCI; 0.1 N, pH 1.1; 2.5 ml/kg body weight) during the inspiratory phase of ventilation. The acid was divided into two aliquots and instilled through a suction catheter into the right and left main bronchus. The injury was considered stable if PaO_2 _was constantly lower than 300 mmHg at an FiO_2 _of 1.0 60 minutes after instillation.

##### Control

Control animals had the same instrumentation for hemodynamic monitoring as those in the AAP group but nothing was instilled into the lungs.

#### Measurements

Measurements were performed at baseline (T_0_) and then 60, 120 and 240 minutes either after lung injury was established or after baseline measurements in the control group.

##### Hemodynamics and gas-exchange

Cardiac output (CO), stroke volume, global ejection fraction, left and right end-diastolic volumes, right ventricular ejection fraction, systemic and pulmonary pressures, extravascular lung water index (ELWI), and intrathoracic blood volume index (ITBI) were measured. Thermodilution measurements were performed in triplicate by the same investigator with 20 ml ice-cold 0.9% saline solution. Arterial and mixed venous samples were collected and immediately analyzed for blood gases (ABL 510, Radiometer, Copenhagen, Denmark).

##### Lung and brain imaging and analysis

CT scans of the lungs were obtained from apex to base during an end-expiratory hold at a PEEP of 5 cm H_2_O (GE Light Speed VCT, GE Medical Systems, München, Germany), thickness 5 mm, interval 0.5 mm, 100 mA, 100 kV). The method used for quantitative image analysis has been described previously [16]. Quantitative analysis of the entire lung was performed to assess lung density (Hounsfield units, HU) and the extent of lung tissue aeration (normal, poor and none). Analysis of individual lung regions was performed by dividing the lung into 10 equidistant horizontal sections along the sagittal axis.

Pulmonary parenchyma with a CT density ranging from -1,000 to -900 HU was classified as overinflated, a range of -900 to -500 HU as normal, -500 to -100 HU as poorly aerated, and -100 to +300 HU as non-aerated (atelectatic).

Three consecutive horizontal sections of the cerebral CT scans starting below the external auditory meatus were analyzed. The brain CT density window was set from -10 to +100 HU. Decreased density (lower HU) represented greater tissue water content, that is, edema, while increased density represented an increase in blood content.

#### Data acquisition

Data recording and analysis were performed using the Modular Intensive Care Data Acquisition System (MIDAS) developed by P. Herrmann and P. Nguyen (Institut für Biomedizinische Technik, Hochschule Mannheim, Germany).

#### Histology

##### Lung, heart, liver and kidney

The tissue samples of the lungs were taken from the dependent part of the right and left lower lobes.

The heart was removed *in toto *and 10 samples each were taken from the right and the left atria and ventricles. Three samples each were taken from the left lobe of the liver and the upper poles of the kidneys.

The samples were fixed in 10% phosphate buffered paraformaldehyde, embedded in paraffin, cut into 1 μM sections and stained with hematoxylin-eosin. The sections were scanned at 25-power then examined in detail at 100 to 250-fold magnification (Olympus BH 2, Hamburg, Germany) and assessed with a semi-quantitative score specific for each organ to grade the extent of inflammation, cell damage and edema (Additional file 1). Apoptosis was detected primarily by morphology. The tissue sections were assessed by two trained observers blinded to the treatment group on two separate occasions each. If an assessment differed between the observers, the section was reassessed and a consensus score was made. The organ scores of the individual samples were averaged for each animal and these averages were used for further statistical analysis.

##### Brain

The brain was removed and fixed in formaldehyde, embedded in paraffin, cut into 1 μm sections and stained with hematoxylin-eosin.

The CA1 and CA2 regions of the hippocampus were studied because they are the regions most vulnerable to ischemic or hypoxic insult [17]. Nuclear pyknosis and eosinophilic degeneration of the cytoplasm were taken as evidence of cell damage. The extent of cell damage was graded using the established score of our Department of Neuropathology: I = individual damaged cells (5 to 10 cells); II = clusters of damaged cells; III = larger regions of damaged cells; IV = severe cell loss. Both right and left hippocampi were examined and the grade of the most severely affected region was used to calculate the brain cell damage score.

### Statistical analysis

Descriptive statistics are expressed as means and standard deviations or medians and interquartile ranges. Non-parametric tests were used for comparative statistics. Changes over time were analyzed globally with the Friedmann-test for each time series and in case of a significant difference followed by the Wilcoxon signed-rank test for paired samples for individual comparisons vs. T0 in order to identify the time points with changes. For comparisons between the two groups, the Mann-Whitney U test (MW-U test) was used as well for the individual time points of the hemodynamic, the CT scans and the histology. Where multiple comparisons were performed, the computed *P*-values were adjusted to control the False Discovery Rate (FDR) using the Benjamini-Hochberg method. The sample size was chosen based on the practicality of the experiments and the required statistical power for the MW-U test. Significant differences at an alpha-level of 0.05 could be detected for the seven animals. The simulated power of the MW-U test at this sample size to detect an average histology score difference of 1 is approximately 70%.

A *P*-value smaller than 0.05 was considered significant. All calculations were performed with Statistica (9.0; StatSoft; Europe, Hamburg, Germany) or with the statistical software R (Robert Gentleman and Ross Ihaka, Statistics Department of the University of Auckland, Free Software Foundation's, GNU General Public License).

The primary endpoints were functional and morphological changes in the studied organs, heart, kidneys, brain and liver. Secondary endpoints were functional and morphological changes in the lungs.

## Results

### Hemodynamics and ICP

Hemodynamic data are shown in Table 1. The hemodynamic variables remained constant in the AAP and control groups during the study period, except for mean pulmonary pressure (mPAP), central venous pressure (CVP), right ventricular ejection fraction (RVEF) and ELWI, which were higher in the AAP group (*P *< 0.05). ICP was comparable at baseline levels and did not change significantly in either group. No hypotensive episodes occurred in any animal at any time.

**Table 1:** Hemodynamic variables

	**T**_**0**_	**T**_**60**_	**T**_**120**_	**T**_**240**_
***HR, min^-1^***				
Control	79.0 ± 23.0	86.0 ± 16.0	96.0 ± 19.0	85.0 ± 20.0
AAP	90.5 ± 19..2	80.7 ± 21.2	69.7 ± 12.9	71.9 ± 9.0
***MAP, mmHg***				
Control	76.0 ± 15.0	81.0 ± 21.0	80.0 ± 14.0	74.0 ± 12.0
AAP	83.9 ± 24.7	73.4 ± 8.0	69.9 ± 5.7	86.5 ± 16.9
***CVP, mmHG***				
Control	6.7 ± 4.0	6.7 ± 4.5	6.2 ± 4.3	7.2 ± 3.7
AAP	8.2 ± 3.3	11.5 ± 3.3	12.5 ± 1.5°	13.6 ± 3.9°
***mPAP, mmHG***				
Control	23.3 ± 4.7	21.1 ± 2.4	21.2 ± 2.1	21.0 ± 4.2
AAP	21.3 ± 6.0	31.4 ± 9.8*°	32.3 ± 8.3*°	30.3 ± 3.1*°
***CO, l.min^-1^***				
Control	7.5 ± 1.5	7.1 ± 1.6	7.3 ± 1.3	6.9 ± 1.0
AAP	8.1 ± 2.4	7.1 ± 2.1	7.4 ± 1.4	6.7 ± 1.2
***SV, ml***				
Control	82.0 ± 18.0	80.0 ± 22.0	78.0 ± 17.0	81.0 ± 20.0
AAP	94.6 ± 17.1	104 ± 29.1	104 ± 28.1	103 ± 37.9
***SVV, %***				
Control	9.7 ± 3.2	10.1 ± 3.6	9.1 ± 2.3	7.6 ± 2.4
AAP	9,9 ± 7.3	9.7 ± 4.8	9.2 ± 4.5	9.8 ± 3.2
***ITBI, ml.m^2^***				
Control	729 ± 125	704 ± 91.0	711 ± 83.0	755 ± 98.0
AAP	793 ± 103	842 ± 172	896 ± 171	902 ± 162
***ELWI, ml/kg***				
Control	9.0 ± 1.9	8.6 ± 1.4	8.5 ± 1.5	8.9 ± 1.1
AAP	8.2 ± 1.9	9.9 ± 1.9	12.5 ± 4.3*	14.5 ± 5.2*°
***GEF, %***				
Control	38.1 ± 7.2	37.5 ± 8.4	37.2 ± 7.6	36.9 ± 8.5
AAP	37.4 ± 2.6	40.7 ± 5.4	37.3 ± 4,.2	39.1 ± 5.4
***RVEF, %***				
Control	36.6 ± 7.1	39.7 ± 15.1	39.1 ± 8.5	35.7 ± 7.6
AAP	41.1 ± 2.1	31.0 ± 7.6*	32.2 ± 5.5*	29.7 ± 6.7*
***RVEDV, ml***				
Control	199 ± 54	194 ± 55	198 ± 49	202 ± 63.0
AAP	210 ± 40	232 ± 71	245 ± 83	293 ± 136
***RHEDV, ml***				
Control	387 ± 110	389 ± 108	400 ± 101	421 ± 114
AAP	415 ± 62.4	437 ± 108	459 ± 96	535 ± 213
***LHEDV, ml***				
Control	557 ± 174	515 ± 122	511 ± 138	550 ± 112
AAP	559 ± 139	581 ± 149	640 ± 209	566 ± 185

Values are the mean (SD) of seven. AAP, acid aspiration pneumonitis; CO, cardiac output; Control, control animals; CVP, central venous pressure; ELWI, extravascular lung water index; GEF, global ejection fraction; HR, heart rate; ITBI, intrathoracic blood volume index; LHEDV, left heart end-diastolic volume; MAP, mean arterial pressure; mPAP, mean pulmonary artery pressure; RHEDV, right heart end-diastolic volume; RVEDV, right ventricular end-diastolic volume; RVEF, right ventricular ejection fraction; SRVEF, right ventricular ejection fraction; SVV, stroke volume variation; V, stroke volume. * *P *< 0.05 vs. T0;°*P *< 0.05 Control vs. AAP

### Lung

#### Gas exchange

Pulmonary gas-exchange was similar in both groups at baseline. PaO_2 _and PaCO_2 _remained constant in control animals but changed significantly in the AAP group (*P *< 0.05) with the greatest impairment after 60 minutes followed by a tendency to improve over the remaining course of the study period (T_240 _vs. T_60_; *P *< 0.05 (Table 2)). Hypoxemia did not occur at any time in any animal.

**Table 2:** Pulmonary gas exchange

FiO_2 _100%	**T**_**0**_	**T**_**60**_	**T**_**120**_	**T**_**240**_
***pHa***				
Control	7.42 ± 0.1	7.36 ± 0.1	7.38 ± 0.1	7.40 ± 0.1
AAP	7.36 ± 0.1	7.24 ± 0.1*	7.27 ± 0.1	7.33 ± 0.1
***PaCO_2_, mmHg***				
Control	41.8 ± 5.0	44.0 ± 8.0	41.8 ± 5.4	34.9 ± 5.4
AAP	43.1 ± 3.0	58.4 ± 12.1*	55.8 ± 10°	51.4 ± 8.5°
***PaO_2_, mmHg***				
Control	486 ± 55	455 ± 78	468 ± 96	470 ± 74
AAP	447 ± 65	261 ± 97*°	332 ± 119*	354 ± 87*^#^°

Values are mean (SD) of seven. AAP, acid aspiration pneumonitis; Control, control animals; FiO_2_, inspired oxygen fraction; PaCO_2_, arterial CO_2 _tension; PaO_2_, arterial oxygen tension; paO_2_/FiO_2_, partial pressure arterial oxygen/fraction inspired oxygen ratio. * *P *< 0.05 vs. T_0_; **^#^**° *P *< 0.05 vs. T_60_, ° *P *< 0.05 Control vs. AAP.

#### Extravascular lung water

The extravascular lung water index (ELWI) remained constant in control animals but increased significantly in the AAP group at 240 minutes (*P *< 0.05).

#### Density, gas content and aeration

##### Total lung analysis

Mean total lung density expressed in HU and percentages of normally, poorly and non-aerated tissue are shown in Table 3. Lung density decreased over time in the control group indicating alveolar recruitment. In the AAP group, lung density increased continuously over time (*P *< 0.05).

**Table 3:** Global lung and brain density and distribution

Lung CT scans		**T**_**0**_	**T**_**60**_	**T**_**120**_	**T**_**240**_
***Hounsfield units***	HU				
*Control*		-673 ± 19.6	-679 ± 25.2	-682 ± 24.5*	-711 ± 25.4*
*AAP*		-649 ± 46.9°	-566 ± 55.3*°	-489 ± 107.3*°	-413 ± 143.2*°
***Normally aerated *** *tissue*	%				
*Control*		83.5 ± 4.0	83.6 ± 5.1	83.7 ± 4.8	84.6 ± 4.0
*AAP*		85 ± 4.3	68.3 ± 6.9*°	57.1 ± 10.6*°	48.6 ± 20.3*°
***Poorly aerated *** *tissue*	%				
*Control*		11.3 ± 3.9	11.2 ± 5.1	10.6 ± 4.5	8.9 ± 4.0*
*AAP*		12.1 ± 4.4	23.7 ± 4.4*°	26.9 ± 7.1*°	25.1 ± 4.4*°
***Non-aerated tissue***	%				
*Control*		2.2 ± 2	2.1 ± 2.4	2.2 ± 2.3	1.9 ± 2.1*
*AAP*		2 ± 0.6	6.9 ± 6.2*°	15.1 ± 10*°	25.6 ± 17.9*°
Brain CT scans					
***Hounsfield units***	HU				
*Control*		32.3 ± 5.4	31.2 ± 5.4	33.4 ± 4.5	32.6 ± 4.7
*AAP*		29.9 ± 3.8	28.7 ± 6.8	29.6 ± 5.7	29.8 ± 5.7

Values are mean (SD) of seven animals per group. AAP, acid aspiration pneumonitis; Control, control animals

* *P *< 0.05 vs. T_0_; ° *P *< 0.05 vs. Control; ^ *P *< 0.05 vs. AAP

The total proportion of normally aerated lung tissue decreased significantly in the AAP animals, while the proportions of poorly aerated and atelectatic lung areas increased significantly (*P *< 0.05) (Table 3).

##### Regional lung analysis

Analysis of the horizontal lung sections showed that the greatest changes in lung density (mean HU) occurred in the dependent regions of the lungs (Additional file 2). There was no significant difference in mean HU in AAP animals compared to control animals in segment 1. Differences were detected in segment 2 after 240 minutes, in segment 3 after 120 minutes and in segments 4 to 10 at all measuring points.

**Figure 1:**
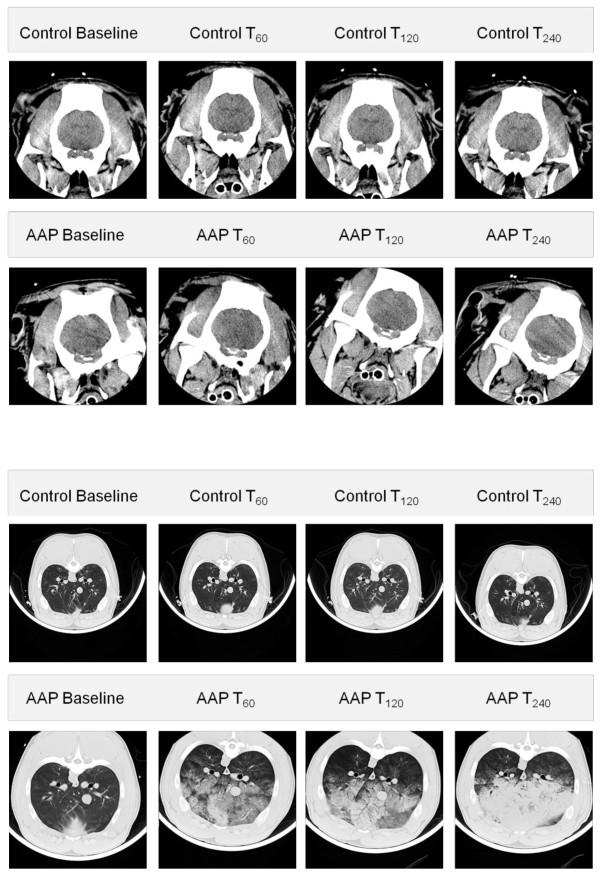
**Cerebral and pulmonary CT scans of the Control and AAP group**. Upper images are cerebral CT scans at baseline, after 60, 120 and 240 minutes in the Control and AAP groups. The lower images are thorax CT scans after 60, 120 and 240 minutes in the Control and AAP groups.

The amount of normally aerated lung tissue between groups was comparable in segment 1 and 2, but significantly different after 60 and 240 minutes in segment 3, from segment 4 to 10 at all measuring points. The extent of poorly aerated tissue was similar in both groups in segment 1, but differed significantly after 240 minutes in segment 2 and at all time points in segments 3 to 10. Non-aerated lung tissue was similar in both groups in segments 1 and 2, significantly increased in the AAP group in segments 3 and 4 after 240 minutes, after 120 minutes in segment 5 and segments 6 to 10 at all measuring points. (Representative CT-images of the lung and brain are presented in Figure 1)

##### Brain density

Mean brain tissue density seen in CT scans did not differ significantly between the two groups (Table 3).

#### Histology: (Table 4, Figures 2, 3 and 4)

**Table 4:** Histology results

a.)						
	**Left Ventricle**		**Left Ventricle Conduction system**		**Right Ventricle**	**Right Ventricle Conduction system**

*Inflammation*						
*Control*	0.8 (0.6/0.9)		0.5 (0.2/0.6)		0.7 (0.5/1.0)	0.3 (0.2/0.4)
*AAP*	1.5 (1.4/1.6)^*^		1.6 (1.5/1.6)*		1.4 (1.2/1.6)*	1.3 (1.1/1.7)*
*Necrosis *						
*Control*	0.2 (0.2/0.4)		--		0.2 (0.1/0.2)	--
*AAP*	0.9 (0.7/1.1)*		--		0.4 (0.3/0.7)*	--

**b.)**						

	**Lung**		**Liver**		**Kidney**	

*Inflammation*						
*Control*	0.3 (0.0/0.5)		0.3 (0.2/0.5)		0.3 (0.3/0.5)	
*AAP*	1.0 (0.8/1.6)*		1.0 (0.3/1.8)*		1.0 (1.0/1.7)*	
*Necrosis *					*proximal tubule*	*distal tubule*
*Control*	0.0 (0.0/0.0)		0.1 (0.0/0.2)		0.2 (0.0/0.3)	0.0 (0.0/0.2)
*AAP*	3.0 (2.7/3.0)*		0.5 (0.2/1.2)		1.1 (0.9/1.7)*	0.5 (0.4/1.2)*
*Edema*	*extraalveolar*	*alveolar*	*extracellular*	*intracellular*	*extracellular*	*intracellular*
*Control*	0.0 (0.0/0.0)	0.0 (0.0/0.2)	0.0 (0.0/0.0)	0.0 (0.0/0.1)	0.2 (0.1/0.3)	0.0 (0.0/0.1)
*AAP*	1.0 (0.9/1.0)*	0.7 (0.7/1.5)*	0.0 (0.0/0.0)	0.0 (0.0/0.9)	1.4 (0.5/1.7)	1.0 (0.5/1.2)

Values are the median (25% quartile/75% quartile) of seven animals per group. AAP, acid aspiration pneumonitis; Control, control animals * *P *< 0.05 control vs. AAP

**Figure 2:**
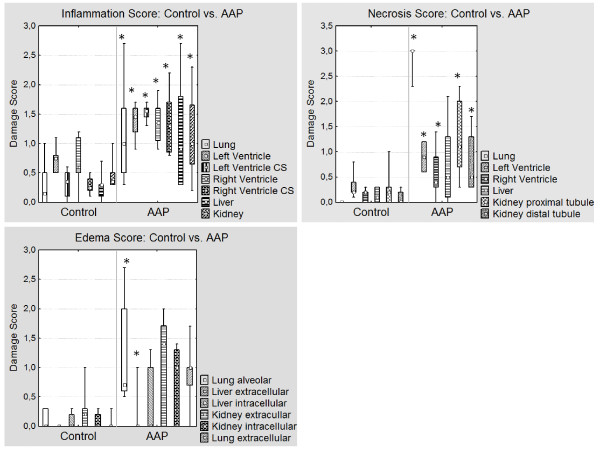
**Histological Score of the Control and AAP group**. Values are presented as median, 25% and 75% quartile, minimum and maximum of seven. Inflammation, necrosis and edema score of seven animals in the Control and AAP group. (Left ventricle CS: left ventricle conduction system; Right ventricle CS: right ventricle conduction system); significance: * *P *< 0.05.

**Figure 3:**
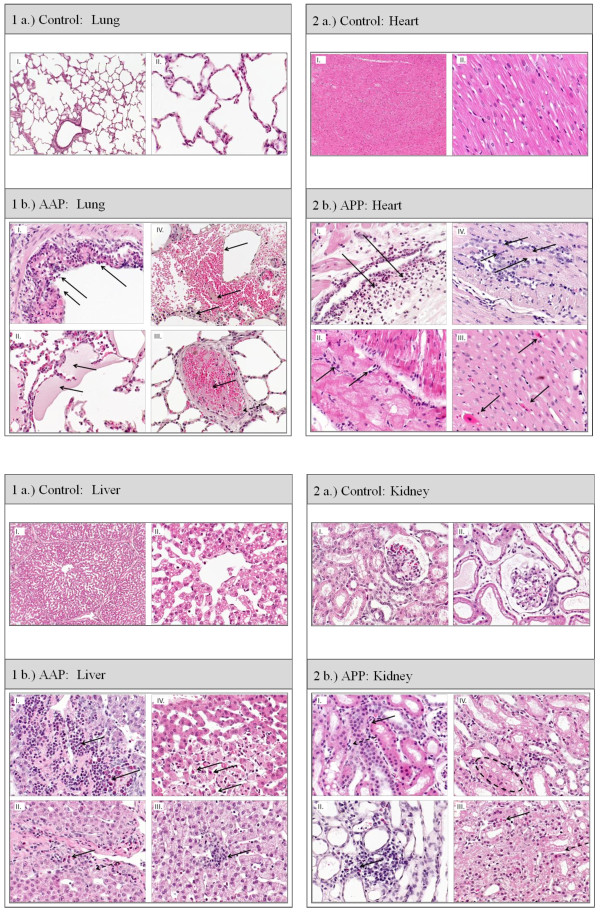
**Histological image of the Control and AAP Group: Lung, Heart, Liver and Kidney (Hematoxylin-eosin)**. **1 a.) **Control lung: I. 25x; normal lung tissue; II. 100x; normal lung tissue **1 b.) **AAP lung: I: bronchiolus with neutrophilic granulocytes and foamy macrophages (←); II: acute congestion/stasis of the lung with intraalveolar exudation and hyaline membranes (←) (shock lung); **III**: pulmonary thrombembolism (←); **IV**: interstitial edema with fibrinoid and hemorrhagic exudation + neutophilic granulocytes, lost of alveolar structures (←). **2 a.) **Control heart: I. 25x. I.) left ventricle: normal cardiomyocytes; II. 100x. I.) left ventricle: normal cardiomyocytes. **2 b.) **AAP heart: I left ventricle: increased number of inflammatory cells, spreading from the vessels to cardiac tissue (→); II: impulse conduction system, lymphocytes, macrophages; spreading from the impulse conduction system to the heart muscle cells (→); III: apoptosis of a cardiomyocyte (←, →); IV: inflammatory cells, cell damage in the center of the image (←, →). **3 a.) **Control liver: I. 25x; Liver: normal liver lobe with regular septum and central vein II. 100x; normal liver lobe with regular septum and central vein. **3 b.) **AAP liver: I: septal region of a portal tract with massive infiltrates of neu-trophilic and eosinophilic granulocytes (←); II: inflammatory cells (←), intracellular edema with swollen hepatocytes (← -), III: inflammatory infiltration in the center (←); IV: central region with cell apoptosis/necrosis and inflammatory cells (←). **4 a.) **Control kidney: I., 25x; regular cortical kidney tissue with a glomerulus in the middle of the image; II. He-matoxylin-eosin, 100x; regular cortical kidney tissue with a glomerulus in the middle of the image. **4 b.) **AAP kidney: I: disseminated inflammatory cells (←), intracellular edema in tubular epithelial cells (← -), II: cluster of inflammatory cells (←), slight interstitial edema; III: granulocytes (←); necrotic tubular epithelial cells (← -); IV: circle: massive intracellular edema.

**Figure 4:**
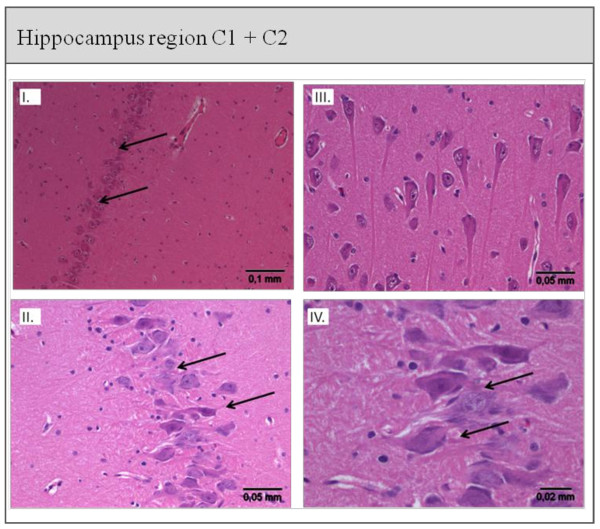
**Histological staining of the Hippocampus region C1 + C2 from the Control (I. + II.) and AAP (III. + IV.) group**. **I. + II**. Hippocampus cells of the C1 and C2 regions from a control animal: cell formation intact and regular, normal neuron density (→). **III. + IV**. Hippocampus cell of the C1 and C2 region from an AAP animal: abnormal cell formation, cell damage, shrinkage of neurons, basophilic neurons with core pyknosis (←). Scoring: I: 5 to 10 damaged cells; II: damaged cell groups; III: unions of damaged cells; IV: massive cell loss.

##### Lung

The inflammation, necrosis and edema scores of the lungs were significantly higher in the AAP group (*P *< 0.05). The tissue samples showed interstitial and intra-alveolar edema, leukocyte infiltration and thickening of the alveolar septa. The pulmonary infiltration consisted primarily of polymorphonuclear neutrophils and spread from the bronchioles to the alveolar ducts. Vascular emboli and erythrocyte extravasation was seen.

##### Heart

The inflammation, necrosis and edema scores of the left and right ventricle as well as the conduction system were significantly higher in the AAP group (*P *< 0.05).

The leukocyte infiltration spread from the conduction system over the endothelium to the cardiac muscle cells. The infiltrating cells consisted mainly of histiocytes/macrophages and lymphocytes. Necrosis and apoptosis of cardiomyocytes were seen. Capillaries were dilated.

##### Liver

The inflammation score of the liver was significantly higher in AAP animals, but there was no difference in the occurrence of necrosis and edema. Infiltration spread from the portal fields (zone 1) over the septa towards the central fields/central veins. Groups of necrotic cells and hepatocytes in lysis were seen in the liver lobules (zone 2), while dilated sinusoids were seen in zone 3. Lymphocytes were the predominant cell type.

##### Kidney

Animals of the AAP group had significantly higher inflammation and necrosis scores in the proximal and distal tubules (*P *< 0.05). The most marked infiltration (primarily lymphocytic) was seen in and around the cortex and particularly in the tubules and collecting tubules. Hyaline and hemoglobin casts were found in the distal and proximal tubules. Anuclear cells and cell lysis were also observed.

##### Brain

The mean cerebral damage scores of the hippocampal regions CA1 and CA2 were 2.0 ± 1.0 in control animals, 2.7 ± 0.8 in AAP animals. Abnormally shaped cells, cell damage, neuronal shrinkage and basophilic neurons with nuclear pyknosis were found, but cell damage did not differ significantly between the groups (*P *= 0.073). However, the cell damage seen in the control animals was most likely due to the intracranial instrumentation.

## Discussion

In this study we observed radiological and histological changes consistent with a systemic inflammatory response to the intrabronchial acid instillation in large animals. Although, gas-exchange and hemodynamics were only moderately impaired, significant damage to the lung, heart, liver and kidney occurred.

Intrabronchial instillation of hydrochloric acid is an established animal model with early direct chemical damage to the alveolar epithelium followed later by inflammation [18,19]. Even unilateral instillation of hydrochloric acid causes bilateral pulmonary damage [20]. In its milder form it has little effect on cardiovascular performance as opposed to models such as infusion of endotoxin or oleic acid into the pulmonary artery [21]. This has the advantage that hypoperfusion and hypoxemia are unlikely to be confounding factors of the observed extra-pulmonary organ injuries in our investigation. Our study animals reliably developed lung injury as shown by the impaired oxygenation with an initial reduction of P_a_O_2_/F_i_O_2 _to under 300 mmHg, but none of the animals experienced hypoxemia. This is similar to the results of Fraisse in his mild injury group of rats after intratracheal acid instillation [22]. Our original hypothesis that circulatory depression and/or hypoxemia are the causative mechanism of extrapulmonary organ damage can probably be discarded, since neither condition occurred in any animal.

Both CT scans and lung histopathology showed significant edema, while the histopathological picture was consistent with an inflammatory reaction with leukocyte infiltration. These changes are the morphological correlates of the observed effects on gas exchange and the increasing extravascular lung water.

Gas exchange was most severely affected 60 minutes after acid instillation (Additional file 3) but returned almost to baseline values during the study period even at low PEEP levels (*P *< 0.05). In contrast, the CT scans showed a continuous, significant increase in density and in poorly and non-ventilated lung areas during the entire study period, primarily in dependent regions (Additional file 3). These results are similar to those seen in a murine model, in which gas exchange recovered but CT scans still showed abnormally aerated lung regions two weeks after the injury [20].

ELWI increased with statistical significance at 120 minutes, while gas exchange had begun to recover from an initial impairment [23]. The evidence of pulmonary edema provided by ELWI paralleled that provided by lung density changes seen in the CT scans. This indicates that moderate aspiration might not be detected or might be underestimated in the clinical setting without a CT scan. Several studies have shown that ELWI reliably indicates lung injury before it can be detected on conventional chest X-ray. ELWI could be a useful parameter to monitor the degree of lung injury after acid aspiration [24,25].

We looked for changes in extrapulmonary organs that could indicate a systemic inflammatory response to the local inflammation in the lungs and provide evidence for organ cross-talk. We found pathological changes in heart, liver, kidneys and brain with the severest lesions in the heart and cardiovascular function indicating that aspiration pneumonitis causes indirect damage to other organs or renders them vulnerable to otherwise innocuous secondary insults. Although not the subject of the present study, there is evidence from studies described below that such extrapulmonary organ damage can impair pulmonary function. One can, therefore, envision an interaction between organs, organ cross-talk, which could act in the manner of a vicious circle.

Acid aspiration led to increased CVP and mPAP with a significant reduction of the right ventricular ejection fraction, while MAP and CO remained constant. These results stand in contradiction to those of Schertel [9], who described significant decreases in MAP, mPAP and CO in a dog model of acid aspiration. Aside from possible species-specific differences, the different responses might be due to the fact that propranolol was administered to the animals in the latter study. This could have reduced cardiac contractility enough to cause the observed effects.

An inflammatory response with cell necrosis was observed in both ventricles with leucocyte infiltration starting in the endothelium and spreading via the conduction system to the endocardium and the myocardium. This involvement of the conduction system may be the reason for the arrhythmias that can occur [26]. We consider the inflammatory response to be the cause of the observed intra- and extracellular edema. This is in accordance with results of other studies that have indicated that lung injury and systemic inflammation are primarily triggered by neutrophils [27,28]. Schertel, on the other hand, also found evidence of myocardial edema, but considered it independent of neutrophil infiltration.

A recently published study showed that lung injury induced by installation of gram-negative bacteria is much more severe when preceded by acid aspiration [29]. This points out the importance of a second hit injury, which might increase multi-organ damage because of the intensification of the primary injury. All examined organs showed a high degree of neutrophilic granulocyte infiltration, which demonstrated that aspiration not only causes a local, but rather a systemic inflammatory response. Particularly the finding of group necrosis, and massive intracellular, as well as extracellular, edema in the tissue samples shows the intensity of the cell damage. Our findings of histocytes and macrophages in all organs confirms a study by Beck-Schimmer, who described that rat alveolar macrophages play an essential role in the inflammatory response after acid-induced lung injury [30]. Similar results have been described in small animals [8,23].

In our study, acute lung injury was associated with significant renal leukocyte infiltration and cell necrosis. This confirms the results of studies with acute lung injury in small animals [31,32] and is in accordance with the clinical observation that lung protective ventilation and the use of an adequate PEEP reduces the severity of renal failure [33,34]. However, it contradicts the results of a study in dogs, in which no evidence of renal injury was seen despite significant acid aspiration lung injury [7]. The reason for this discrepancy is not immediately evident, but could reflect a species-specific difference. Of note in this context is a study by Vieira, which showed that renal damage not only affects overall mortality but also impairs weaning from the ventilator [35]. This would indicate and be consistent with organ cross-talk.

Because our study design required intracranial instrumentation of all study animals, a certain amount of cerebral damage was necessarily induced by this measure in the control animals. This cerebral damage would tend to decrease the difference in the damage scores between the two groups. This could be the reason for the fact that although the brain pathology scores showed a strong tendency towards a statistically significance difference between the control and AAP groups (*P *= 0.073), our significance criterium was not met. This interpretation is supported by the fact that we did observe cell damage in all other studied organs, and by the results published by Aaltonen [36], who described pathological changes in the hippocampus induced by meconium aspiration in piglets. Hemodynamic parameters were stable and no hypoxemia occurred in their study animals, as in ours, and they detected damage to the hippocampus [36]. Previous studies have shown that the hippocampus is highly sensitive to any kind of trauma [37] or systemic inflammation [38].

It remains to be determined whether lung protective ventilation, that is, limiting tidal volume, plateau pressure and the use of an adequate PEEP [34,39,40] in the early phase after aspiration could also prevent or alleviate the systemic inflammatory response and thus not only protect the lung but also extra-pulmonary organs.

### Limitations

In this study, only the early effects on lung density, leukocyte infiltration and cell damage as well as edema in the lung, heart, liver kidney and brain were studied. Cardiac and pulmonary function but not that of the other organs was studied. Another limitation is the lack of immunohistochemical methods, which might have been helpful in understanding the underlying mechanisms of the observed changes.

## Conclusions

Aspiration affects not only gas exchange and lung tissue but also has a multi-systemic impact on organ function. Multi-organ impairment and histological damage characterized by neutrophil infiltration occurred in the absence of hypoxemia and circulatory instability ruling out these as causative factors. ELWI is a sensitive bedside parameter for monitoring the course of lung injury after acid aspiration. Further studies are necessary to elucidate the pathways and interactions following acute aspiration induced lung injury.

## Key messages

• Acid aspiration pneumonitis causes extrapulmonary organ injury.

• Heart, liver, kidneys and brain show varying degrees of inflammation, edema and necrosis.

• The primary pulmonary damage is inflammation and edema.

• The edema is best quantified by the extravascular lung water index (ELWI).

## Abbreviations

μM: micrometer; AAP: acid aspiration pneumonitis; BW: body weight; CA1 and CA2: regions in the hippocampus; CO: cardiac output; CO_2_: carbon dioxide; CT: computed tomography; CVP: central venous pressure; ELWI: Extravascular Water Index; F: French; FDR: false discovery rate; FiO_2_: fraction of inspired oxygen; GEF: global ejection fraction; HCI: hydrochloric acid; HR: heart rate; HU: Hounsfield units; I:E: inspiratory: expiratory ratio; ICP: intracranial pressure; ITBI: Intrathoracic Blood Volume Index; Kg: kilogram; LHEDV: left heart end-diastolic volume; MAP: mean arterial pressure; MIDAS: Modular Intensive Care Data Acquisition System; mPAP: mean pulmonary arterial pressure; MW-U test: Mann-Whitney U test; n: number; PEEP: positive end expiratory pressure; RHEDV: right heart end-diastolic volume; RVEDV: right ventricular end-diastolic volume; RVEF: right ventricular ejection fraction; SDC: supplemental digital content; SV: stroke volume; SVV: stroke volume variation; VT: tidal volume.

## Competing interests

The authors declare that they have no competing interests.

## Authors' contributions

JFH, PP, PH and MQ planned and designed the study. JFH, PS, PH and PP performed the measurements and analyzed the data. WB was responsible for the brain histology and CP was responsible for the histology of the other organs. FS provided funding for the histology studies and allowed us to use his laboratory facilities and technicians. All authors (JFH, PP, PS, PH, WB, CP, FS, TAC and MQ) participated in the analysis and interpretation of the results. The final manuscript was drafted by JFH, TAC and PS and was discussed, read and approved for publication by all participating authors.

## Supplementary Material

Additional file 1 **Histopathological score**. Histopathological semi quantitative organ-specific scores of lung, myocardium, kidney and liver used to assess extent of inflammation, cell damage and edema. Click here for file

Additional file 2 **Mean Hounsfield units in the Control and AAP group**. The figure shows the lung density measured in Hounsfield Units (HU) from lung segment 1 (non-dependent lung segment) to segment 10 (dependent lung segment) at baseline and after 60, 120 and 240 minutes in two groups. Click here for file

Additional file 3 **Arterial oxygen content and computer tomography of the AAP lungs from baseline to 240 minutes**. The figure shows the mean Hounsfield Units (HU), the percentage of normally, poorly and non-aerated tissue as well as the arterial oxygen content at baseline, and after 60, 120 and 240 minutes in AAP animals. Click here for file
