# Supplementary material for: Effects of pulmonary acid aspiration on the lungs and extra-pulmonary organs: a randomized study in pigs
Source: Crit Care. 2012 Mar 1;16(2):R35. doi: 10.1186/cc11214 (PMC3681347; doi:10.1186/cc11214)
Supplement: Additional file 1 — Histopathological score. Histopathological semi quantitative organ-specific scores of lung, myocardium, kidney and liver used to assess extent of inflammation, cell damage and edema. [file cc11214-S1.PDF]

Additional Files:

Additional File 1:

Histopathological Score:

Semiquantitative Scoring:

Lung ***(per low power field (lpf, 100x)):***

Inflammation:
0: no significant inflammatory cells
1: < 25 granulocytes, lymphocytes, macrophages
2: 25- 50 granulocytes, lymphocytes, macrophages
3: > 50 granulocytes, lymphocytes, macrophages
Necrosis:
0: no necrosis
1: <5 scattered necrotic cells
2: 5-25 scattered or clustered necrotic cells
3: >25 clustered necrotic cells
Edema:
Interstitial:
0: no edema
1: ¼ of the interstitial tissue is affected
2: ½ of the interstitial tissue is affected
3: fully affected interstitial tissue with severe fibrin and hemorrhagic exudates
Alveolar:
0: no edema
1: ¼ of alveolar space
2: ½ of alveolar space
3: fully affected alveolar spaces with severe fibrin and hemorrhagic exudates

---

Myocardium (per low power field (lpf, 100x)):

Inflammation:
0: no significant inflammatory cells
1: < 10 granulocytes, lymphocytes, macrophages
2: 10-50 granulocytes, lymphocytes, macrophages
3: > 50 granulocytes, lymphocytes, macrophages
Necrosis:
0: no necrosis
1: < 3 scattered apoptotic/necrotic cardiomyocytes
2: < 5 scattered apoptotic/necrotic cardiomyocytes
3: > 5 clustered apoptotic/necrotic cardiomyocytes
Edema: Not evaluated because of overlap with necrotic changes

---

Kidney (10 proximal and 10 distal tubules were evaluated):

Inflammation:
0: no significant inflammatory cells
1: < 5 granulocytes, lymphocytes, macrophages
2: 5-25 granulocytes, lymphocytes, macrophages
3: > 25 granulocytes, lymphocytes, macrophages
Necrosis:
0: no necrosis
1: < 3 necrotic tubular epithelial cells
2: < 10 necrotic tubular epithelial cells
3: > 10 necrotic tubular epithelial cells

Edema:

Extracellular:

0:	no edema
1:	1/4 of interstitial renal tissue
2:	1/3 of interstitial renal tissue
3:	1/2 of interstitial renal tissue, optionally hemorrhage

Intracellular:

0:	no edema
1:	< 2 swollen epithelial cells per tubule
2:	< 5 swollen epithelial cells per tubule
3:	> 1/2 of all epithelial cells per tubule

Liver (per low power field (lpf, 100x)):

Inflammation:

0:	no significant inflammatory cells
1:	< 15 clustered granulocytes, lymphocytes, macrophages
2:	15-50 clustered or disseminated granulocytes, lymphocytes, macrophages
3:	> 50 clustered or disseminated granulocytes, lymphocytes, macrophages

Necrosis:

0:	no necrosis
1:	<5 scattered necrotic hepatocytes
2:	5-10 scattered or clustered necrotic hepatocytes
3:	>10 scattered or clustered necrotic hepatocytes

Edema:

Extracellular:

0:	no edema
1:	< 1/4 of interstitial tissue and sinusoides
2:	< 1/3 of interstitial tissue and sinusoids, compression of hepatocellular plates
3:	> 1/3 of interstitial tissue and sinusoids, compression of hepatocellular plates

Intracellular:

0:	no edema
1:	< 5 swollen hepatocytes
2:	5-30 swollen hepatocytes
3:	> half of all hepatocytes swollen
