# Supplementary material for: Effects of pulmonary acid aspiration on the lungs and extra-pulmonary organs: a randomized study in pigs
Source: Crit Care. 2012 Mar 1;16(2):R35. doi: 10.1186/cc11214 (PMC3681347; doi:10.1186/cc11214)
Supplement: Additional file 2 — Mean Hounsfield units in the Control and AAP group. The figure shows the lung density measured in Hounsfield Units (HU) from lung segment 1 (non-dependent lung segment) to segment 10 (dependent lung segment) at baseline and after 60, 120 and 240 minutes in two groups. [file cc11214-S2.PDF]

Additional Files:

Additional File 2:

Mean Hounsfield units in the Control and AAP group.

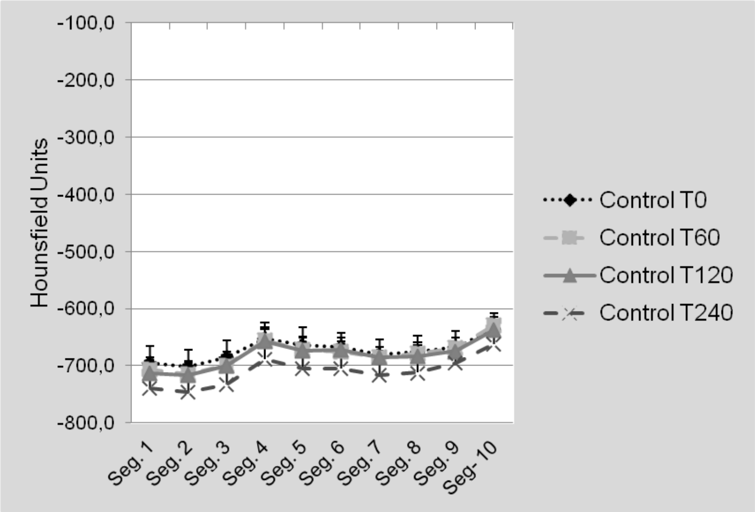

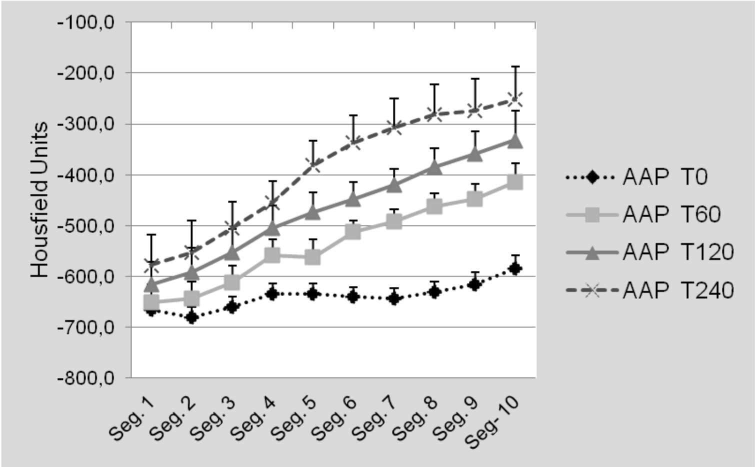

Mean (+ SE) lung density (Hounsfield units) in Control (upper figure) and AAP (lower figure) from Segment 1 (non-dependent) to Segment 10 (dependent). Control: Control; AAP: Acid aspiration pneumonitis. TO: baseline; T60: 60 minutes; T120: 120 minutes; T240: 240 minutes.
