# Supplementary material for: Effects of pulmonary acid aspiration on the lungs and extra-pulmonary organs: a randomized study in pigs
Source: Crit Care. 2012 Mar 1;16(2):R35. doi: 10.1186/cc11214 (PMC3681347; doi:10.1186/cc11214)
Supplement: Additional file 3 — Arterial oxygen content and computer tomography of the AAP lungs from baseline to 240 minutes. The figure shows the mean Hounsfield Units (HU), the percentage of normally, poorly and non-aerated tissue as well as the arterial oxygen content at baseline, and after 60, 120 and 240 minutes in AAP animals. [file cc11214-S3.PDF]

Additional Files:

Additional File 3:

Arterial oxygen content and computer tomography of the AAP lungs from baseline to 240 minutes

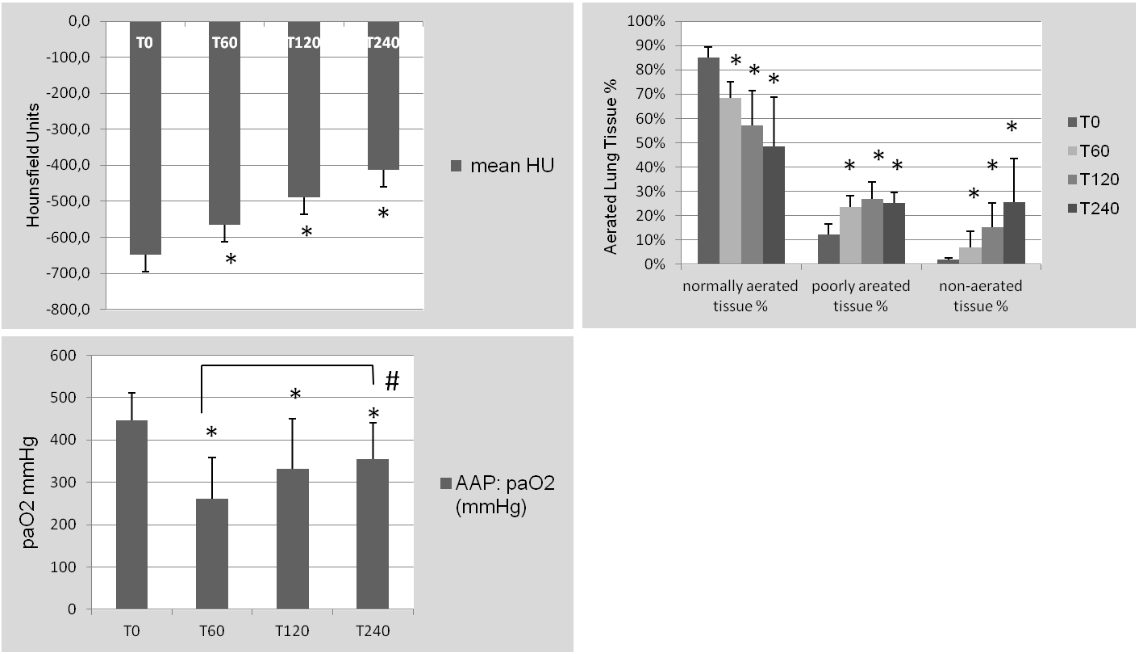

Values are presented as mean with standard deviation. Left upper figure: mean HU: mean Hounsfield Units, T0: baseline, T60 : 60 minutes, T120 : 120 minutes, T140 : 240 minutes; imaging of the HU over the experiment. Right upper figure: imaging of the normally, poorly and non-aerated lung tissue in percent over the experiment. Left lower figure: paO_2: partial arterial oxygen content; T0: baseline, T60 : 60 minutes, T120 : 120 minutes, T140 : 240 minutes ; Imaging of the paO_2. significance: * significant ($p < 0,05$) in relation to baseline; # significant in relation to T60.
